# Lightweight genome viewer: portable software for browsing genomics data in its chromosomal context

**DOI:** 10.1186/1471-2105-8-344

**Published:** 2007-09-18

**Authors:** Jeremiah J Faith, Andrew J Olson, Timothy S Gardner, Ravi Sachidanandam

**Affiliations:** 1 Bioinformatics Program, Boston University, USA; 2 Cold Spring Harbor Laboratory, USA; 3 Department of Biomedical Engineering, Boston University, USA

## Abstract

**Background:**

Lightweight genome viewer (lwgv) is a web-based tool for visualization of sequence annotations in their chromosomal context. It performs most of the functions of larger genome browsers, while relying on standard flat-file formats and bypassing the database needs of most visualization tools. Visualization as an aide to discovery requires display of novel data in conjunction with static annotations in their chromosomal context. With database-based systems, displaying dynamic results requires temporary tables that need to be tracked for removal.

**Results:**

lwgv simplifies the visualization of user-generated results on a local computer. The dynamic results of these analyses are written to transient files, which can import static content from a more permanent file. lwgv is currently used in many different applications, from whole genome browsers to single-gene RNAi design visualization, demonstrating its applicability in a large variety of contexts and scales.

**Conclusion:**

lwgv provides a lightweight alternative to large genome browsers for visualizing biological annotations and dynamic analyses in their chromosomal context. It is particularly suited for applications ranging from short sequences to medium-sized genomes when the creation and maintenance of a large software and database infrastructure is not necessary or desired.

## Background

Genome browsers are the primary tools for the visualization of raw genomic sequence data and annotations. Typically, these software systems are web-based and present an image with "tracks" of information that describe the underlying genome sequence. The tracks include features such as SNPs, ESTs, linkage-disequilibrium, and splice variants. Navigation through these annotations is done by zooming and scrolling along the track and the underlying sequence information.

Initially, most organisms with complete genomes had their own custom-built genome browser software [1-3]. More recently, there has been a push towards feature-rich species-generic genome browsers that can be reused for new genomes. The result is a small number of high quality genome browsers that are used across many species [3-7]. All of these browsers use a large set of annotations, which are input into a relational database. A collection of scripts then read the information for the genome region a user wants to view and presents the annotations corresponding to that region.

The large software systems used by genome browsers often require specialized knowledge for installation and maintenance. The requirement of a relational database complicates the genome browsers' applicability in dynamic contexts that change frequently. In addition, running a full-fledged genome browser on a personal computer is not trivial.

Here we present *lightweight genome viewer *(lwgv), a genomic sequence annotation visualizer that requires only a single text file and executable to run. This simplicity and independence from a database backend facilitates the dynamic creation of genome views based on user-chosen analyses. lwgv allows "include" files, which provide an object-oriented, plug-n-play, architecture for managing tracks and building text files for more complex viewer applications. We have successfully used lwgv to visualize RNAi oligos on their corresponding genes [8], to present a linkage disequilibrium map of chromosome 19 [9], and to display feature annotations for the GeneSeer [10]. We also present a new application of lwgv to dynamically visualize changes in gene expression along a genome using any combination of the over 500 prokaryotic microarrays available in the *Many Microbe Microarrays Database *(M^3D^). lwgv is an ideal tool for the presentation of dynamic analyses and sequence annotations without resorting to the creation and maintenance of a large database and software infrastructure.

## Implementation

**Figure 1:**
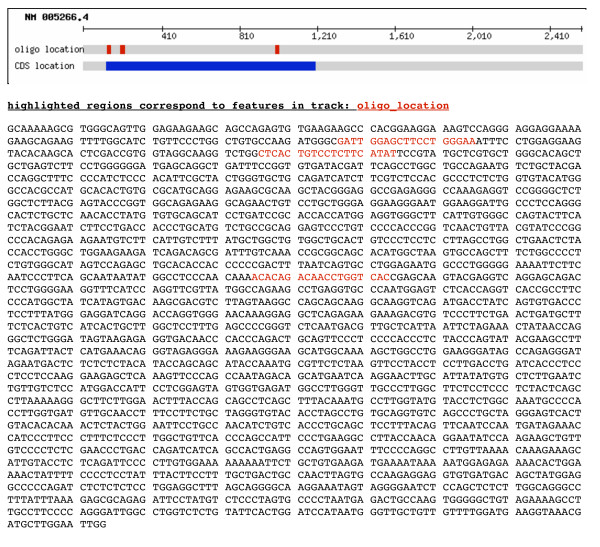
A screenshot of the results from a siRNA design visualization demonstrates how lwgv represents sequence features on tracks. In this case, the features of interest are the coding sequence (CDS) of the gene being used for RNAi and the location of the designed oligos on that CDS. lwgv also allows the sequence itself to be displayed and colored according to the information in the track.

**Figure 2:**
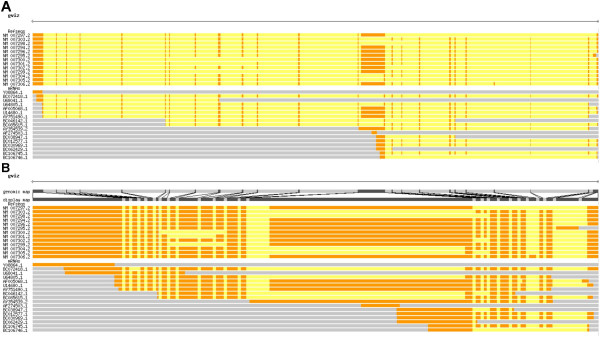
Example of the trackCorrelate function to help visualize splice variants. RefSeq and other mRNAs from the human gene BRCA1 are shown aligned to the genome (a) and after compressing introns (b). After compression, it is much easier to see the different isoforms and, for example, discover that mRNAs BC072418.1 and AF005068.1 are not represented among the RefSeqs.

lwgv runs as a web-based CGI program. Genome features are represented as color-coded tracks on a web browser, and detailed information about each feature can be shown by "mousing-over" them (Figure 1). These features are described in a text-file written in a simple descriptive language. In addition, we offer translators that accept standard annotation formats including BED, WIG, PSL, GFF, and GenBank. Each track, or feature within a track, can have its own unique color, and features across tracks can have lines connecting them to show, for example, the boundaries of homologous sequences across two species or to compare alternative splice sites (Figure 2). In addition to tracks, the sequence viewer can represent numerical information along a genome using line plots, smooth line plots (using cubic splines), or histograms. Basic properties like image width, track height, and navigation buttons are all configurable. Commonly used feature sets and configuration parameters can be stored in separate files and included into an annotation file with an "#include" statement to prevent regenerating the same features in contexts where only part of the analysis data is dynamic.

## Results and discussion

### lwgv as a traditional genome browser

Although lwgv is not as feature rich as database-driven genome browsers, it is sufficiently fast to be used in place of a genome browser in many contexts where the number of tracks and features is not too large (e.g. less than 10 tracks with less than 10000 features each). For example, lwgv was used to display a linkage disequilibrium map of human chromosome 19 [9,11]. lwgv is a good replacement for traditional genome browsers when quick setup is needed and the visualization demands require only simple tracks and graphs. Larger software packages, such as the UCSC Genome Browser [12], should be used when more advanced browsing features are needed (e.g. expandable tracks, the ability to dynamically add/remove tracks, and the ability to navigate large genomes by clicking on regions of a chromosome image).

### lwgv as a short sequence viewer

Most software applications for visualizing short DNA sequences are standalone-applications that are only available commercially or are devoted to a specific task such as restriction enzyme digestion [13]. lwgv is well suited for visualizing annotations of short stretches of DNA. For example, we use lwgv to show the location of the RNAi knockdown clones from the Hannon-Elledge shRNA libraries [8,14]. For this task, it is only necessary to show individual genes and the location of each shRNA designed for those genes. With lwgv, this task can be done by reading the available shRNAs for a particular gene from the database of all shRNAs and generating the corresponding annotations such as exon boundaries and shRNA binding sites into a temporary file to be read by lwgv. This dynamic approach allows one to update the shRNA database without having to sync a second database for a genome viewer.

lwgv is particularly well suited to dynamically display a user's analysis of a particular region of DNA. We previously developed a web application where biologists can design their own RNAi oligos [15]. lwgv provides a simple way to show the locations of the RNAi oligo designs on the user's sequence (Figure 1). For traditional genome browsers, this would require either generating (and subsequently deleting) a new database or table for each user or developing a lot of workaround code to allow the genome browser to operate from a database that has many discontinuous sequences from different species. With lwgv, the user's sequence can be visualized by generating the appropriate temporary file with their sequence and the location of the siRNA oligos on their sequence. These temporary files can be deleted when they are past a pre-determined expiration date.

### lwgv as a dynamic microarray analysis tool

**Figure 3:**
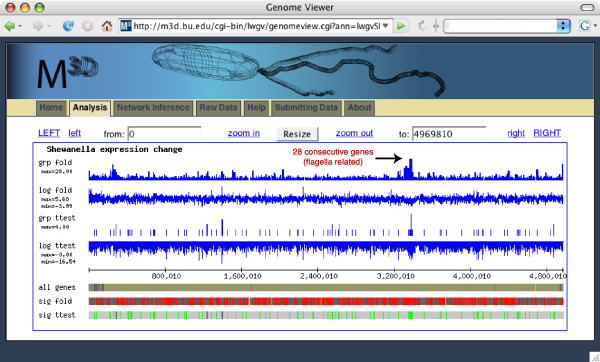
lwgv is used with the *Many Microbe Microarrays Database *to allow users to dynamically display expression changes in their chromosomal context. In this example, significant expression changes between *E. coli *cells grown in rich media and *E. coli *cells grown in rich media with norfloxacin antibiotic are shown with lwgv. In this chromosomal context, it is immediately clear that several large regions of the genome have significantly changed expression levels between these two conditions. For example, over 28 consecutive genes and intergenic regions related to flagella have a significant fold change (track *grp fold)*. These significantly changed genes are displayed on track *sig fold*.

Common microarray analysis procedures yield lists of genes, whose expression changes significantly in response to an environmental or genetic perturbation. The functional role for most of these expression changes is typically unknown, and the often-large number of changed genes hinders human interpretation of their role. In many species, genes with similar functional roles often exhibit chromosomal proximity and therefore operate as a co-expressed module, even when part of distinct operons and transcription units [16,17]. To facilitate the sharing, discovery, and analysis of expression data in a genome localization context, we created an lwgv application where users can dynamically choose any two sets of microarray experiments in M^3D ^and view gene expression changes in their chromosomal context (Figure 3). M^3D ^includes Affymetrix microarray compendia for multiple microbes including *S. oneidensis*, *E. coli*, and *S. cerevisiae*, and it also provides visualization and data download tools [18,19]. lwgv is also packaged with a script that allows any expression data in the commonly used GPR format to be visualized in a genome context.

## Conclusion

lwgv is a lightweight genome browser that can be used in small-scale projects and individual labs. Scientists and laboratories with little computing infrastructure can use lwgv since it does not require databases or other software.

## Availability and requirements

lwgv is distributed under the GPL license.

**Project name: **lwgv

**Project homepage: **

**Operating systems: **linux and mac os x

**Programming languages: **C

**Other requirements: **apache, cgic, gd graphics library, lex (flex), yacc (bison)

**Any restrictions to use by non-academics: **none

lwgv is distributed as a source code tarball and installs with the standard unix "./configure" and "make" commands. Details about installing lwgv, writing tracks, and customizing the output can be found in the manual and README files distributed with the software.

## Competing interests

The author(s) declares that there are no competing interests.

## Authors' contributions

JJF and RS developed the initial source code. AO wrote parsers for converting other genome browser annotations into the lwgv format. All authors contributed bug fixes and minor additions to the software. All authors contributed to writing the manuscript. All authors read and approved the final manuscript.
